# Perspectives in transvaginal sonography for the diagnosis of adenomyosis

**DOI:** 10.3389/fmed.2025.1533648

**Published:** 2025-06-19

**Authors:** Xiao-Ling An, Jun Zhang, Xiao-Chun Yun, Xiao-Hui Lei, Jing Zhao, Xiao-Hua Yang, Mei-Mei Liu

**Affiliations:** ^1^Department of Ultrasound, New ChangAn International Maternity Hospital, Xi’an, China; ^2^Department of Utrasound, Angel Women’s and Children’s Hospital Affiliated to Northwest University, Xi’an, China; ^3^Department of Ultrasound, Xi’an Gaoxin Hospital, Xi’an, China

**Keywords:** adenomyosis, transvaginal sonography, transvaginal ultrasound, perspective, diagnosis

## Abstract

Adenomyosis is a common gynecological condition affecting women of reproductive age, posing significant diagnostic challenges due to its diverse clinical presentations. This extended opinion study critically reviews the diagnostic methods for adenomyosis, with a focus on transvaginal sonography (TVS), a widely used non-invasive imaging technique. The study evaluates the effectiveness, limitations, and clinical applicability of TVS, while exploring the role of histopathological confirmation when non-invasive methods are insufficient. Advancements in TVS, including three-dimensional ultrasound and color Doppler, have enhanced diagnostic precision, particularly in assessing uterine morphology and blood flow. Additionally, artificial intelligence integration offers potential to further optimize diagnostic accuracy and efficiency. While histopathological examination remains the gold standard, its use is often impractical in patients who do not undergo hysterectomy. This study provides a comprehensive overview of the current status of TVS in diagnosing adenomyosis, analyzing its accuracy, strengths, and limitations across diverse patient populations. Results suggest that TVS is a reliable diagnostic tool, though its accuracy improves when combined with magnetic resonance imaging, especially in complex cases. Ongoing research is needed to refine TVS capabilities and identify non-invasive alternatives to histopathological confirmation, improving accessibility and diagnostic efficiency.

## 1 Introduction

Adenomyosis, a significant gynecological condition, is characterized by the infiltration of endometrial tissue into the uterine muscle layer, leading to a variety of symptoms including severe pain and excessive menstrual bleeding (1). These symptoms significantly impact the quality of life of the affected women. Current data suggest that 20–40% of women in their reproductive years may be affected by adenomyosis (2). However, the actual prevalence may be higher due to the variability of symptoms and the complexity involved in diagnosing the condition accurately.

Among the various diagnostic techniques available, transvaginal sonography (TVS) is recognized for its non-invasive nature, practicality, and cost-effectiveness, making it the preferred diagnostic method (3). TVS utilizes high-frequency sound waves to create detailed images of the uterus and surrounding pelvic organs, thereby offering a non-invasive approach to diagnosing adenomyosis. TVS has an advantage over abdominal ultrasound because it provides clearer images due to its proximity to the uterus (4). This diagnostic technique is instrumental in confirming the presence of adenomyosis and in evaluating the lesions’ size, location, and extent—key factors in devising an appropriate treatment plan (5).

The diagnostic approach to adenomyosis via TVS has seen significant advancements, particularly with the incorporation of three-dimensional (3D) ultrasound and color Doppler techniques. These innovations have greatly enhanced the assessment of blood flow dynamics and the structural details of lesions, thus improving the sensitivity and specificity of diagnoses (4, 6). Moreover, the adoption of the Morphological Uterus Sonographic Assessment (MUSA) guidelines within the TVS framework has standardized the evaluation process, ensuring more accurate and reproducible diagnoses (7–9). The MUSA system includes key sonographic features—subendometrial echogenicity, myometrial heterogeneity, and adenomyoma—which are strongly correlated with histopathological findings and widely used in clinical practice (7–9). These features are selected for their consistent diagnostic relevance and ease of identification, making them essential for distinguishing adenomyosis from other uterine conditions (7–9). While the MUSA system includes several additional features, the three highlighted are the most frequently observed and diagnostically significant. Other features, such as myometrial thickness changes and cystic areas, are less common or more variable, limiting their utility in routine clinical practice.

Advancements in medical imaging, particularly TVS, have substantially improved the diagnostic process for adenomyosis, a gynecological condition often characterized by complex and elusive diagnostic challenges. This extended opinion study critically assesses the role of TVS as the primary non-invasive diagnostic tool for adenomyosis, focusing on its clinical efficacy, diagnostic accuracy, and inherent limitations. Additionally, it explores the potential integration of artificial intelligence (AI) to enhance the diagnostic performance and efficiency of TVS in identifying adenomyosis (10, 11).

Although histopathological examination remains the gold standard for adenomyosis diagnosis, its application is frequently impractical, particularly in non-surgical patients. Consequently, TVS has emerged as an increasingly essential tool in clinical practice. This study synthesizes current research findings to provide a comprehensive evaluation of TVS, highlighting its strengths and weaknesses and comparing its effectiveness to traditional diagnostic approaches. The analysis underscores the growing significance of non-invasive diagnostics and positions TVS as a crucial method for addressing some of the challenges in diagnosing adenomyosis.

The primary aim of this study is not to introduce new scientific data but to offer a consolidated perspective on the current status of TVS in adenomyosis diagnosis. By synthesizing existing evidence, this study aims to provide actionable insights and evidence-based recommendations to inform clinical practice and direct future research, particularly in refining non-invasive diagnostic methodologies for adenomyosis.

## 2 Search strategy and study selection

A systematic search was conducted across the Web of Science and PubMed databases, encompassing publications from their inception through September 30, 2024. The search utilized specific keywords, including “adenomyosis” and “transvaginal sonography,” to identify studies relevant to the diagnostic role of TVS in adenomyosis. Only studies published in English were considered to ensure comprehensive inclusion of international research.

Eligibility criteria were confined to prospective and retrospective studies published in peer-reviewed journals. Included studies needed to focus specifically on the diagnostic application of TVS in adenomyosis. Studies were excluded if they addressed unrelated aspects of TVS and adenomyosis, were non-clinical in nature, or were duplicates.

The initial search yielded a total of 198 articles. After applying the predefined inclusion and exclusion criteria, 176 studies were excluded for reasons of irrelevance or failure to meet eligibility requirements. Subsequently, 22 full-text articles were reviewed in detail, of which 14 studies satisfied all inclusion criteria and were retained for the final analysis (Table 1).

**Table 1:** Clinical studies assessing the diagnostic accuracy of TVS for adenomyosis.

Study	Patients (n)	Prevalence (%)	Diagnostic accuracy (%)	Comparison	Findings
Zannoni et al. 2020 (20)	78	33.3	Sensitivity: 77.0; Specificity: 96.0; PPV: 89.0; NPV: 90.0.	Comparison with histopathology	The question mark sign and TVS uterine tenderness are valuable diagnostic tools for adenomyosis.
Atzori et al. 1996 (21)	175	NR	Sensitivity: 86.6; Specificity: 96.2; PPV: 68.4; NPV: 98.0.	Comparison with histopathology	TVS is an important and advanced tool in the diagnosis of diffuse adenomyosis.
Rasmussen et al. 2019 (22)	110	29	2D-TVS Sensitivity: 72; Specificity: 76; PPV: NR; NPV: NR. 3D-TVS Sensitivity: 69; Specificity: 86; PPV: NR; NPV: NR.	Comparison with histopathology	For diagnosing inner myometrium adenomyosis, 3D-TVS is as accurate as 2D-TVS, with junctional zone irregularities aiding diagnosis. Combining 2D and 3D features may offer a more objective diagnosis, valuable for clinical practice and research.
Tellum et al. 2018 (23)	100	NR	Sensitivity: 85; Specificity: 78; PPV: NR; NPV: NR.	Comparison with histopathology	Findings could assist clinicians in interpreting the varied ultrasonographic appearance of adenomyosis.
Sharma et al. 2015 (24)	100	NR	Sensitivity: 95.6; Specificity: 93.4; PPV: 88.6; NPV: 97.6.	Comparison with histopathology	Color Doppler, by assessing blood flow impedance parameters (PI, RI, Vmax) in arteries associated with uterine lesions, significantly improves the differentiation and diagnosis of leiomyoma and adenomyosis beyond 3D TAS and TVS morphological criteria.
Bazot et al. 2001 (25)	120	33	Sensitivity: 65.0; Specificity: 97.5; PPV: 92.8; NPV: 88.8.	Histopathology correlation and MRI comparison	TVS matches MRI in diagnosing adenomyosis in women without myoma, but MRI is preferable for those with concurrent leiomyoma.
Kepkep et al. 2007 (26)	70	37.1	Sensitivity:80.8; Specificity: 61.4; PPV: 84.4; NPV: 68.6.	Comparison with histopathology	Subendometrial echogenic linear striations, globular configuration, and myometrial cysts on transvaginal ultrasound are key indicators of adenomyosis, with subendometrial linear striations being the most accurate diagnostic marker.
Sun et al. 2010 (27)	213	39.9	Sensitivity: 87.1; Specificity: 60.1; PPV: 59.2; NPV: 87.5.	Correlation with histopathology	Subendometrial echogenic linear striations, heterogeneous myometrial echotexture, and anterior–posterior asymmetry on transvaginal ultrasonography are indicative of adenomyosis, with linear striations being the most accurate diagnostic feature.
Exacoustos et al. 2011 (28)	72	44.4	2D-TVS Sensitivity:75; Specificity: 90; PPV: 86; NPV: 82. 3D-TVS Sensitivity:91; Specificity: 88; PPV: 85; NPV: 92.	Correlation with histopathology	The coronal section from 3D-TVS allows for precise assessment of the junctional zone, whose alteration accurately diagnoses adenomyosis.
Hanafi M. 2013 (29)	163	75.4	Sensitivity: 84.6; Specificity: 43.4; PPV: 75.6; NPV: 57.5.	Correlation with histopathology	TVS is a valuable noninvasive diagnostic method for identifying leiomyoma and combined adenomyosis with leiomyoma, being sensitive for both conditions but lacking specificity for adenomyosis alone.
Di Donato et al. 2015 (30)	50	48	Sensitivity: 83; Specificity: 88; PPV: 87; NPV: 85.	Correlation with histopathology	A novel ultrasonographic sign, prevalent in women with adenomyosis and exhibiting high specificity, holds potential for diagnosing suspected cases of adenomyosis and distinguishing it from other uterine disorders.
Luciano DE et al. 2013 (31)	54	66.6	Sensitivity: 92; Specificity: 83; PPV: 99; NPV: 71.	Correlation with histopathology	3D TVS accurately locates adenomyosis in the uterine walls, but its diagnostic accuracy can be affected by changes in the junctional zone due to endometrial ablation and medical therapy.
Dueholm et al. 2001 (32)	106	21	Sensitivity: 68; Specificity: 65; PPV: 42; NPV: 85.	Correlation with histopathology, comparison with MRI.	Combining MRI and TVS offers high accuracy in excluding adenomyosis, but low specificity may necessitate further investigation of positive cases, with junctional zone thickness measurement potentially improving MRI diagnoses.
Reinhold et al. 1996 (33)	119	24	Sensitivity: 89; Specificity: 89; PPV: 71; NPV: 96.	Histopathology correlation and MRI comparison.	TVS was found to be as accurate as MRI for diagnosing uterine adenomyosis, and using a junctional zone thickness of ≥12 mm could further enhance the diagnostic accuracy of MRI.

TVS, transvaginal sonography; MRI, magnetic resonance imaging; NPV, negative predictive value; PPV, positive predictive value; NR, not reported.

## 3 Diagnostic standards and methods

The diagnosis of adenomyosis necessitates a multifaceted approach, encompassing clinical assessment, detailed imaging studies, and sometimes histopathological examination (1, 11, 12). Below is a detailed exploration of each step in the diagnostic process.

Initial diagnosis begins with a comprehensive evaluation of the patient’s clinical history and symptoms (1). Particular attention is paid to reports of heavy menstrual bleeding, severe menstrual pain, and chronic pelvic pain, which are suggestive of adenomyosis (1). A gynecological examination may also provide indicative signs, such as a uniformly enlarged, tender uterus.

TVS serves as the primary imaging technique for the non-invasive diagnosis of adenomyosis (11, 12). It involves the use of high-frequency sound waves to create detailed images of the uterus, allowing for the observation of characteristic signs such as heterogeneous myometrial texture, myometrial cysts, and areas of acoustic shadowing, all of which indicate abnormal infiltration of endometrial tissue into the myometrium (11, 12). TVS’s ability to detect these aberrations makes it an invaluable diagnostic tool, though its sensitivity and specificity can be influenced by the operator’s expertise and the ultrasound equipment’s quality (11). Despite these challenges, TVS remains a cornerstone diagnostic tool for adenomyosis, particularly in patients not requiring hysterectomy, offering a non-invasive and widely accessible approach to diagnosis (11).

The importance of imaging techniques, especially TVS, is critical in the early diagnosis of adenomyosis, particularly in adolescents and young women. In this age group, the clinical presentation of adenomyosis is often subtle, and symptoms can be easily confused with or misattributed to other common gynecological conditions (13, 14). As a widely accessible and non-invasive modality, TVS is commonly employed as the first-line diagnostic tool in these populations. However, its ability to detect more extensive or atypical lesions is limited, especially in cases where the presentation is less typical or more complex (13, 14). In these scenarios, complementary imaging modalities such as magnetic resonance imaging (MRI) are essential to provide a more accurate and comprehensive diagnostic evaluation.

Histopathological examination has long been regarded as the gold standard for the definitive diagnosis of adenomyosis, especially when surgical intervention, such as hysterectomy, is indicated (15, 16). This involves the analysis of uterine tissue obtained through hysterectomy, where the presence of endometrial glands and stroma within the myometrium, extending beyond the normal endometrial-myometrial junction, serves as a diagnostic hallmark for adenomyosis (15, 16). However, the limitations of current biopsy techniques, which may not consistently provide reliable or conclusive results—particularly in cases where tissue access is restricted—necessitate a reconsideration of the role of histopathological confirmation as an essential criterion for diagnosing adenomyosis, especially when diagnosis is based on imaging findings (16).

In this context, TVS presents as a highly relevant diagnostic tool, particularly for patients who are not candidates for hysterectomy or prefer non-surgical alternatives (11, 12). TVS provides a non-invasive, highly valuable diagnostic approach, facilitating the detection of hallmark features of adenomyosis, including subendometrial echogenicity, myometrial heterogeneity, and adenomyomas (11). Given the challenges involved in obtaining histopathological confirmation in these patients, TVS plays a pivotal role in clinical practice by offering an accurate, timely, and non-invasive diagnostic alternative (12). Therefore, while histopathological examination remains essential in select cases, its necessity should be reconsidered for patients who are not candidates for hysterectomy, where TVS has demonstrated significant diagnostic reliability.

## 4 Diagnosing adenomyosis via TVS

### 4.1 Fundamental principle of ultrasound imaging

TVS operates on the principle of employing high-frequency sound waves to elucidate the structural and pathological characteristics of the uterus and adjacent anatomical entities. This diagnostic modality is inherently non-invasive and provides a real-time visual assessment of gynecological health (4, 17).

**Generation and transmission of ultrasound waves**: The process begins with the transvaginal introduction of an ultrasound probe, which emits sound waves in the high-frequency range, typically between 5 and 9 MHz (11, 18). These frequencies are chosen for their optimal balance between penetration depth and resolution, crucial for gynecological imaging.

**Interaction of sound waves with tissues**: As these sound waves traverse through various tissue layers, they encounter interfaces between tissues of differing acoustic impedances (12, 18). Acoustic impedance is a property that reflects how much resistance an ultrasound wave encounters as it passes through a tissue. When waves transition between tissues with varying impedances, part of the wave is reflected back toward the probe, creating echoes.

**Echo detection and image formation**: The probe acts as both a transmitter and receiver of these ultrasound waves. The returning echoes are captured by the probe and relayed to the ultrasound machine, which employs sophisticated algorithms to convert the time delay and intensity of these echoes into digital images (18).

**Tissue differentiation and image resolution**: The principle that sound waves travel at different velocities in various media, coupled with their variable reflection intensities, enables the delineation of tissue boundaries and internal structures with remarkable detail. The variation in echo patterns is interpreted by the system to generate a cross-sectional image of the uterus and surrounding structures on a monitor. This image formation is facilitated by the differential absorption, reflection, and transmission of the ultrasound waves by various tissues, allowing for a detailed anatomical and sometimes functional assessment (12, 18).

**Clinical implications**: The ability of TVS to provide detailed images stems from the fundamental properties of ultrasound waves and their interaction with biological tissues (11, 12, 18). This technique is invaluable in diagnosing and monitoring a wide array of gynecological conditions, including abnormalities of the uterine lining, fibroids, and ovarian cysts (5, 11). Moreover, TVS serves as a critical tool in fertility assessments and early pregnancy evaluations. In the context of adenomyosis, TVS is essential for evaluating the involvement of different regions of the myometrium, which is directly linked to symptom severity and presentation (1, 5, 11, 19). When the internal myometrium is affected, patients commonly experience more severe symptoms, such as menstrual irregularities, dysmenorrhea, and pelvic pain (19). On the other hand, when the external myometrium is involved, the symptoms tend to be less specific and often present as chronic pelvic discomfort, which can be mistakenly attributed to other gynecological conditions (19). This variability in symptom presentation highlights the necessity of comprehensive imaging to assess the full extent of the disease.

### 4.2 Advantages of TVS in adenomyosis

**Detailed uterine assessment**: TVS is exceptionally proficient at visualizing the uterine architecture, enabling the evaluation of ectopic endometrial tissue infiltration and myometrial thickening—key features of adenomyosis (20). It facilitates an detailed examination of the uterus’s size, shape, and the presence of adenomyotic cysts with high precision.

**Clinical significance**: Given its ability to detect nuanced changes in the uterus’s internal structure, TVS is invaluable for the early detection and management of adenomyosis. Its utility in assessing the disease’s impact on uterine morphology makes it a preferred initial diagnostic tool in clinical practice (20). While histopathological confirmation remains the gold standard in some cases, TVS offers a valuable, non-invasive alternative, especially for patients who are not candidates for hysterectomy. The lack of a reliable biopsy technique in such cases underscores the need for further research into non-invasive diagnostic methods.

## 5 Application of TVS in diagnosing adenomyosis

**Figure 1:**
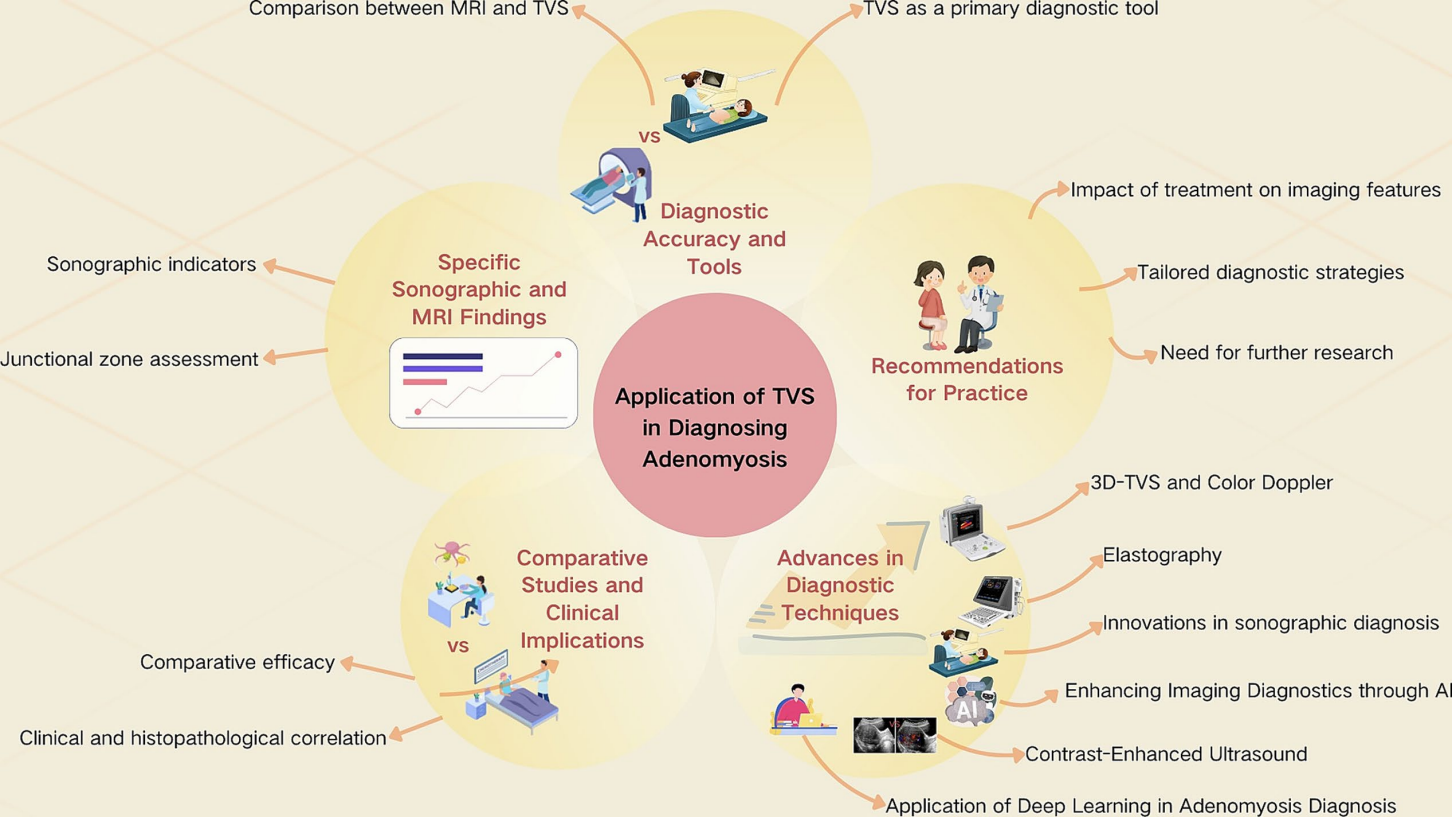
The application of TVS in diagnosing adenomyosis.

The clinical research on the use of TVS for diagnosing adenomyosis is outlined as follows (21–34) (Table 1; Figure 1).

### 5.1 Diagnostic accuracy and tools

**TVS as a primary diagnostic tool**: TVS has proven effective in diagnosing adenomyosis, with specific sonographic signs like the “question mark sign” and uterine tenderness being highly indicative of the condition (21). Advanced techniques such as 3D-TVS enhance the diagnostic accuracy, particularly in evaluating the junctional zone and myometrial echotexture (23, 29) (Table 1; Figure 1).

**MRI Vs. TVS**: MRI offers superior specificity, especially in cases with concurrent leiomyoma, making it a preferred method in complex cases (26). However, TVS holds comparable accuracy for adenomyosis diagnosis in the absence of myoma, highlighting the technique’s value in a broad clinical setting (33, 34) (Table 1; Figure 1).

### 5.2 Specific sonographic and MRI findings

**Sonographic indicators of adenomyosis**: Studies have identified specific TVS features associated with adenomyosis, including subendometrial echogenic linear striations, myometrial cysts, and the globular configuration of the uterus (27, 28). These findings underscore the importance of detailed sonographic evaluation in diagnosing adenomyosis (Table 1; Figure 1).

**Junctional zone assessment via MRI and TVS**: Precise measurement of the junctional zone thickness, especially when utilizing MRI, enhances the diagnostic accuracy for adenomyosis. A thickness of ≥12 mm has been suggested as a significant indicator of the condition (34) (Table 1; Figure 1).

### 5.3 Comparative studies and clinical implications

**Comparative efficacy of diagnostic modalities**: Some studies directly compare the efficacy of TVS and MRI, noting that while MRI may offer higher specificity, TVS remains a crucial diagnostic tool due to its accessibility and cost-effectiveness (26, 33). The combination of both modalities can maximize diagnostic accuracy, particularly for excluding adenomyosis (33) (Table 1; Figure 1).

**Impact of clinical and histopathological correlation**: Correlating sonographic and MRI findings with histopathological outcomes reinforces the diagnostic criteria and enhances the understanding of adenomyosis’s varied presentation. This correlation is critical for refining diagnostic approaches and improving patient management (21–34) (Table 1; Figure 1).

### 5.4 Advances in diagnostic techniques

**3D-TVS and color Doppler utilization**: The adoption of 3D-TVS and the incorporation of color Doppler for assessing blood flow impedance offer significant improvements in differentiating adenomyosis from leiomyoma and other conditions, providing a more nuanced approach to diagnosis (23, 26, 29, 32) (Table 1; Figure 1).

**Innovations in sonographic diagnosis**: Novel sonographic signs and the detailed assessment of uterine morphology contribute to the evolving landscape of adenomyosis diagnosis, aiding clinicians in distinguishing adenomyosis from other uterine pathologies with greater specificity (31) (Table 1; Figure 1).

**Contrast-enhanced ultrasound (CEUS)**: CEUS represents a significant advancement in the ultrasonographic diagnosis of adenomyosis (35, 36) (Figure 1). This technique utilizes microbubble contrast agents that enhance the echogenicity of the blood, allowing for superior visualization of vascular patterns within the uterus (37). CEUS has been shown to be particularly effective in distinguishing adenomyosis from other uterine anomalies such as fibroids (36). The clarity in perfusion patterns provided by CEUS is critical in complex diagnostic scenarios where conventional ultrasound may provide limited information. This modality has demonstrated potential in improving diagnostic accuracy by offering detailed views of blood flow dynamics that are indicative of adenomyosis.

**Elastography**: Elastography, both in its quantitative and qualitative forms, offers a unique perspective in the diagnosis of adenomyosis by evaluating the stiffness of the uterine muscle (37–39) (Figure 1). Adenomyosis typically results in increased tissue stiffness due to fibrosis and myometrial hyperplasia. Qualitative elastography visually maps the elasticity of tissues, providing a color-coded representation, whereas quantitative elastography quantifies tissue stiffness, offering specific numerical values (39). Recent studies have highlighted the utility of elastography in enhancing the diagnostic accuracy of adenomyosis (38). This technique is particularly valuable in cases where the sonographic appearance of adenomyosis mimics that of normal myometrial tissue or fibroids, thus providing additional diagnostic clarity that is crucial for accurate assessment (37).

### 5.5 Application of deep learning in adenomyosis diagnosis

Deep learning has transformed the landscape of medical imaging diagnostics by enabling more accurate and efficient analysis (Figure 1). Recent research has developed several deep learning models tailored specifically for the diagnosis of adenomyosis through enhanced interpretation of imaging data (40). These models leverage large, annotated datasets to train algorithms capable of identifying intricate patterns undetectable by the human eye. The integration of such models with conventional TVS techniques has markedly improved diagnostic accuracy and speed, factors crucial in optimizing patient outcomes. This integration not only facilitates a more streamlined diagnostic process but also ensures the reliability of the diagnoses provided to patients. We have included references to pivotal studies that demonstrate the effectiveness of deep learning models, underscoring their growing importance in the diagnostic toolkit for adenomyosis.

### 5.6 Enhancing imaging diagnostics through AI

The integration of AI into medical imaging represents a transformative advancement, particularly for the diagnosis of complex gynecological conditions such as adenomyosis (41) (Figure 1). AI technologies, utilizing machine learning and deep learning frameworks, augment the diagnostic capabilities of conventional imaging modalities such as TVS and MRI (42). These AI-enhanced tools excel in pattern recognition, enabling them to detect subtle variations in tissue structures that are often challenging for traditional methods to discern. For example, AI algorithms applied to MRI data can accurately distinguish adenomyosis from other uterine anomalies like fibroids by analyzing textural and structural differences in imaging scans (43). This precision is achieved through the algorithm’s ability to learn from vast datasets of annotated images, improving its diagnostic accuracy over time. Similarly, AI can automate and refine the analysis of TVS images, such as measuring the junctional zone thickness or identifying the specific echogenic features associated with adenomyosis, thereby enhancing both the speed and accuracy of diagnoses.

Integrating these AI technologies not only aids radiologists in making more precise evaluations but also fosters a more individualized approach to patient management. The application of AI in imaging for adenomyosis is not just about technological enhancement but also about enabling a shift toward more personalized and responsive healthcare.

### 5.7 Recommendations for practice

**Tailored diagnostic strategies**: Given the variability in diagnostic accuracy and the specificity of findings across different imaging modalities, a tailored approach, considering patient history and clinical presentation, is recommended for diagnosing adenomyosis (24, 30) (Table 1; Figure 1).

**Need for further research**: Ongoing studies focusing on the refinement of diagnostic criteria and the development of predictive models are essential for advancing the diagnosis and management of adenomyosis, ensuring patients receive the most accurate diagnosis and appropriate care (24) (Table 1; Figure 1).

**Impact of treatment on imaging features**: Treatment for adenomyosis, including medical therapies like hormonal treatment or surgical interventions, can lead to notable changes in the ultrasonographic features of the disease (44–46). Hormonal therapies, for example, may reduce the size of adenomyomas or decrease myometrial heterogeneity, resulting in improved echogenicity and a more homogeneous myometrial appearance on ultrasound (45, 46). Similarly, surgical interventions such as uterine artery embolization or hysterectomy typically lead to a significant reduction in adenomyotic tissue, which may normalize the myometrial texture and reduce characteristic sonographic signs (44). These treatment-induced changes underscore the need for follow-up imaging to assess the efficacy of the therapy and monitor the progression or resolution of the disease over time. Regular post-treatment imaging is critical to evaluate the long-term effectiveness of therapeutic interventions and to detect potential recurrences.

## 6 Research advancements in TVS technology

Recent advancements in TVS, particularly the integration of high-frequency ultrasound, 3D ultrasound, and color Doppler ultrasound, have significantly improved the diagnostic accuracy of adenomyosis. These technological innovations not only enhance diagnostic precision but also provide deeper insights into the pathophysiology of adenomyosis.

### 6.1 High-frequency ultrasound

Enhanced Resolution for Early Detection: Utilizing sound waves at elevated frequencies, high-frequency ultrasound transcends traditional imaging limitations, offering superior resolution. This technological leap permits the detailed visualization of minuscule lesions and ectopic endometrial cysts within the myometrium, pivotal for the early identification of adenomyosis and delineation of lesion extent (47).

### 6.2 3D ultrasound

**3D uterine imaging**: The evolution of 3D ultrasound technology from conventional two-dimensional imaging facilitates a comprehensive 3D reconstruction of the uterus (48). This advancement significantly augments the understanding of uterine architecture in adenomyosis, enabling precise assessment of lesion depth and spatial distribution, which is invaluable for surgical planning and prognosticating therapeutic outcomes.

### 6.3 Color Doppler ultrasound

**Vascular insights through blood flow analysis**: Color Doppler ultrasound, by visualizing and quantifying blood flow, offers essential insights into the vascular characteristics of adenomyosis lesions. The identification of heightened blood flow within affected areas serves as a hallmark of adenomyosis, assisting clinicians in differentiating adenomyosis from other gynecological pathologies (49).

### 6.4 Advancements in diagnostic standards and methods

**Quantitative diagnostic criteria**: The amalgamation of these innovative ultrasound technologies has catalyzed the revision of diagnostic criteria for adenomyosis. Current research endeavors aim to quantify adenomyosis indicators, such as uterine muscle layer volume alterations via 3D ultrasound and specific blood flow metrics in lesions through color Doppler ultrasound (29). These quantitative approaches enhance the objectivity and replicability of adenomyosis diagnosis, offering a foundation for more precise patient assessment.

### 6.5 Recent advances in the diagnosis of adenomyosis

In the rapidly evolving field of gynecological diagnostics, several innovative technologies have significantly advanced the accuracy and non-invasiveness of adenomyosis diagnosis (33, 38–40, 42, 43). High-resolution MRI techniques now provide unprecedented detail of the uterine structure, improving the detection and characterization of adenomyosis (33). Additionally, advanced elastography methods have emerged, offering quantitative assessments of tissue stiffness that correlate with adenomyotic changes within the uterus (38, 39).

The burgeoning field of molecular diagnostics also offers promising prospects. Researchers are identifying specific biomarkers that could potentially enable earlier and less invasive detection of adenomyosis compared to traditional methods. Concurrently, the integration of AI with imaging analysis has matured, yielding algorithms that can differentiate adenomyosis from other conditions such as uterine fibroids with enhanced accuracy and efficiency (40). These technological strides not only expedite the diagnostic process but also center it more closely around the patient’s needs, significantly reducing the reliance on invasive diagnostic procedures (42, 43).

## 7 Diagnostic accuracy and limitations

The diagnostic prowess of TVS in identifying adenomyosis has been substantiated through various studies, underscoring its capability to provide high-resolution insights into the uterus’s internal structure. However, the intrinsic limitations and the potential for misdiagnosis necessitate a nuanced analysis of its accuracy and the exploration of strategies to ameliorate these challenges.

### 7.1 Diagnostic accuracy of TVS in adenomyosis

**Empirical evidence of TVS efficacy**: Research has underscored the diagnostic accuracy of TVS and MRI against histopathological benchmarks in adenomyosis detection (29, 33). The advent of 3D ultrasound and Doppler techniques has markedly refined the capability to distinguish adenomyosis from other uterine anomalies, like leiomyoma, by offering intricate details on blood flow and structural anomalies (29).

### 7.2 Recognizing limitations and challenges in diagnosis

**Inherent diagnostic limitations**: The effectiveness of TVS is not without limitations. Variables such as patient anatomy, uterine positioning, and coexisting conditions, notably fibroids, can obscure or distort ultrasound imagery, thus complicating the diagnostic accuracy. The diversity in adenomyosis’s sonographic presentation amplifies the risk of diagnostic inconsistencies absent histopathological correlation (30). The differentiation between adenomyosis and other uterine pathologies continues to pose a significant challenge, emphasizing the necessity for advanced proficiency in ultrasound interpretation.

### 7.3 Enhancing diagnostic precision

**Advanced imaging integration**: Adopting 3D ultrasound and color Doppler technologies enriches the diagnostic framework (29, 32). These modalities correlate strongly with histological analyses, particularly in evaluating the junctional zone, thereby enhancing diagnostic accuracy.

**Educational and training interventions**: Familiarity with adenomyosis’s sonographic hallmarks is imperative for improving TVS specificity (27, 28). The proficiency and experience of the sonographer emerge as critical factors in mitigating operator-dependent discrepancies, underscoring the value of specialized training and education.

**Comprehensive diagnostic approach**: A holistic diagnostic approach, integrating TVS findings with clinical symptomatology and patient history, optimizes the diagnostic process (24). This patient-centered strategy enhances the specificity and relevance of adenomyosis diagnosis, aligning imaging findings with clinical insights.

## 8 Future outlooks

### 8.1 Advancements in ultrasound technology

**Innovations in high-resolution imaging**: The advent of high-resolution transducers and sophisticated software for image analysis heralds a new era in ultrasound diagnostics. The proven effectiveness of 3D ultrasound and color Doppler technologies in delineating adenomyosis from conditions such as leiomyomas underscores the value of these innovations (25). Future enhancements in transducer technology and imaging software are anticipated to further refine diagnostic accuracy, potentially facilitating earlier detection and more nuanced characterizations of adenomyosis.

### 8.2 Integration of AI and machine learning in ultrasound diagnostics

**Harnessing data analytical capabilities**: The application of AI and ML in ultrasound diagnostics represents a frontier with vast potential (50). By processing and analyzing extensive imaging datasets, AI and ML algorithms can uncover patterns not readily discernible to human operators. While specific applications in adenomyosis remain to be fully realized, the general principle suggests a promising avenue for developing automated detection algorithms. These technologies could standardize the identification of sonographic features characteristic of adenomyosis, thereby mitigating observer variability and enhancing diagnostic consistency.

### 8.3 Optimizing diagnostic standards and processes

**Standardization of sonographic criteria**: Establishing uniform sonographic criteria for diagnosing adenomyosis is imperative for ensuring diagnostic accuracy across various clinical contexts (33). Initiatives should focus on adopting standardized criteria, akin to those proposed by pioneers in the field, to facilitate consistent and reliable diagnoses.

**Enhancing sonographer expertise**: Developing specialized training programs aimed at adenomyosis recognition via ultrasound is crucial for minimizing diagnostic variability. The proficiency of the sonographer is a pivotal factor in the accuracy of ultrasound diagnostics, emphasizing the need for targeted educational efforts.

**Multidisciplinary diagnostic approaches:** Adopting an integrated diagnostic strategy that encompasses transvaginal ultrasound findings, clinical assessments, surgical insights, and histopathological analyses offers a comprehensive perspective on adenomyosis (29). This holistic approach enhances the depth and accuracy of the diagnostic process.

**Research and development in AI and ML**: Continued exploration into the application of AI and ML within ultrasound diagnostics is essential (51). Focused research on refining these technologies for practical deployment in identifying adenomyosis and other gynecological disorders is needed to realize their full diagnostic potential.

## 9 Summary

TVS is a key diagnostic tool for adenomyosis, valued for its non-invasiveness, convenience, and cost-effectiveness. Advances in imaging technologies, such as high-frequency ultrasound, 3D imaging, and Doppler ultrasound, have enhanced diagnostic accuracy. The integration of AI and ML is expected to further improve detection precision and consistency, leading to more tailored treatments. Clinicians should adopt these innovations, prioritize ongoing professional development, and refine diagnostic practices to improve patient care. Staying current with emerging technologies and research is essential for enhancing diagnostic and therapeutic outcomes. The combination of advanced ultrasound and AI/ML holds great potential to improve adenomyosis management and patient quality of life, offering more accurate, personalized care in gynecology.

## Data Availability

The original contributions presented in the study are included in the article/supplementary material, further inquiries can be directed to the corresponding author.
